# What type of rural? Assessing the variations in life expectancy at birth at small area-level for a small population province using classes of locally defined settlement types

**DOI:** 10.1186/1471-2458-14-162

**Published:** 2014-02-13

**Authors:** Mikiko Terashima, Judith Read Guernsey, Pantelis Andreou

**Affiliations:** 1 Department of Community Health & Epidemiology, Dalhousie University, 5790 University Avenue, Halifax, Nova Scotia B3H 1V7, Canada

**Keywords:** Small area analysis, Health inequalities, Rural health, Life expectancy at birth, Gender

## Abstract

**Background:**

Although efforts have been made to articulate rural–urban health inequalities in recent years, results have been inconsistent due to different geographical scales used in these studies. Small-area level investigations of health inequalities will likely show more detailed pictures of health inequalities among diverse rural communities, but they are difficult to conduct, particularly in a small population region. The objectives of this study were: 1) to compare life expectancy at birth for females and males across small-areas classified by locally defined settlement types for a small province in Canada; 2) to assess whether any of the settlement types explains variations in life expectancy over and above the extent of socioeconomic disadvantage and social isolation; and 3) to examine variations in life expectancies within a (larger) area unit used as the basis of health inequality investigations in previous studies.

**Methods:**

Seven settlement types were determined for the ‘community’ units based on population per-kilometre-road density and settlement forms. Mean life expectancies at birth for both genders were compared by settlement type, both for the entire province and within the Halifax Regional Municipality—the province's only census designated metropolitan area, but also contains rural settlements. Linear regression analyses were conducted to assess the statistical associations between life expectancy and the settlement types, adjusting for indicators of community-level deprivation.

**Results:**

While types of communities considered as ‘rural’ generally had lower life expectancy for both genders, the effects of living in any settlement type were attenuated once adjusted for socioeconomic deprivation and social isolation. An exception was the village and settlement cluster type, which had additionally negative effects on health for females. There were some variations observed within the Halifax Regional Municipality, suggesting the importance of further investigating a variety of health and disease outcomes at smaller area-levels than those employed in previous studies.

**Conclusions:**

This paper highlighted the importance of further articulating the differences in the characteristics of rural at finer area-levels and the differential influence they may have on health. Further efforts are desirable to overcome various data challenges in order to extend the investigation of health inequalities to hard-to-study provinces.

## Background

In the last 10 to 15 years, an increasing number of studies have started investigating inequalities in health and health behaviours between rural and urban regions. However, the answers to the ’rural or urban’ question have been mixed [1-3]. Riva and colleagues [1] pointed out that the inconsistencies in the results of comparisons may be attributed to a wide range of health measures used for comparisons, and the level of geographical detail used to define rural areas. Indeed, health measures vary from incidence and prevalence of chronic diseases, injury, suicide and health behaviours to life expectancy, premature mortality, and health services utilization [3-9]. The definitions of rural (and urban) used and size of areas attached to the definitions in empirical studies also vary greatly [10,11].

In more recent years, studies comparing various health outcomes not only between urban and rural, but also among different degrees of rural, have emerged [1,6,12,13]. In Canada, a classification of rurality called Metropolitan Influence Zone (MIZ) [14] has increasingly been used to assess the inequalities in health within rural regions [4,8,9,15,16]. MIZ is defined at the Census Subdivision (CSD) level which usually represents municipalities, and is often used in combination with Census definitions of urban (i.e., Census Metropolitan Area and Census Agglomeration Area [CMA/CA]) to show an urban–rural continuum. Some studies linked CSDs with ‘communities’ [4]. MIZ classifies CSDs into zones (Strong-, Moderate-, Weak- and No-MIZ) based on the levels of influence by CMA/CA, measured by the proportion of commuters and geographical distance to large urban centres of 10,000 or more people [8].

Using MIZ, a national study showed that life expectancies at birth for both women and men in Strong-MIZ are higher than those of other rural or urban areas. Prevalence of asthma for women was lower in Weak-MIZ than other areas including urban, while higher diabetes prevalence was observed in Weak- and No-MIZ. Leisure time and physical activity levels were higher in Strong- or Weak-/No-MIZ than Moderate-MIZ, though not as high as urban. There were greater proportions of smokers in Strong- and Weak-MIZ than urban areas, while the proportions were smaller than Moderate- and No-MIZ [8,15]. In a provincial study, [4] life expectancy at birth was higher in Strong-MIZ than others, though the differences were not large across the urban–rural continuum. General gradients in accounts of motor vehicle injury-related mortality, respiratory and circulatory related disease mortality, and suicide rates were observed in both the national and provincial studies. Two studies of health service utilization patterns [9,16] highlighted the heterogeneity in frequency of different service use (e.g., family physicians, specialists, mental health, surgeons, hospital stays) between rural and urban and among different MIZ areas.

MIZ based definitions of rurality provide finer details of differences in health and social characteristics related to health than county or equivalent size areas, yet are big enough to ensure statistical power for analysis involving sparse Canadian rural. As an administrative area unit, CSDs are also convenient in linking many census based data [4] and allow cross provincial comparison with consistent geographical units.

However, there is a notable weakness in studies using MIZ. The geographical area level attached to the definition is municipality, which is still too large a scale to ascertain important differences within the area unit. Commuters to large urban centres are unlikely to be evenly distributed but rather concentrated in specific geographical location(s) within. Municipality-level analysis does not allow investigations of the influence by the social process and relationships unique to smaller settings like communities or neighbourhoods [17,18].

The challenge of understanding the effects of rurality on health is often compounded by its strong correlations with socioeconomic characteristics (i.e., rural communities tend to have less income earning and employment opportunities). Empirical evidence is not consistent as to whether rurality contributes to health over and above socioeconomic conditions in these areas [5,8,19-21]. Assessing the effects of living in different settlement types that “correspond to ‘the use of space to provide the setting of interaction’” [17] relevant to health, while isolating out the socioeconomic conditions of the respective areas, will likely add to our understanding of what it is about being rural that influences health.

This study had three specific objectives. First, it compared life expectancy at birth for females and males across small-area units, classified by 7 different settlement types locally defined for Nova Scotia, a province in Canada. This study utilized area units that were designed in consultation with local planning officials to correspond to generally perceived ‘community’ identities and encompass both urban and rural areas. These are substantially smaller than area units employed in previous studies, and had previously not been used as a basis of population health studies due to their difficulty in geographically linking health data. Second, it assessed whether any of the 7 settlement types explain variations in life expectancy for females and males across the province, above and beyond the proportion explained by two measures of community-level deprivation. Third, it examined whether there was a significant variation between communities within Halifax Regional Municipality (HRM)—the province’s largest municipality containing most urban and rural settlement types.

### Study context

The Province of Nova Scotia has under 1 million residents (n = 913,465 based on 2006 Census). It is the second smallest province in Canada with an area of about 55,000 km^2^. According to the MIZ definition, about 40% of the population live in rural areas. Few studies have been conducted that compared population health statuses or health determinants across the whole province at smaller area-level than CSDs [9,22,23]. HRM is the only census designated metropolitan area (CMA) in Nova Scotia. The distance between the metropolitan centre and the community furthest away within HRM is about 120 km, with some incongruent settlements in between. Calculation of health statuses and social determinants for this municipality as one community, therefore, will likely mask some important differences within it.

## Methods

### Data

Mortality data from Nova Scotia Vital Statistics (2003–2007) were obtained to calculate life expectancy at birth for females and males (n = 40,694). Canadian census (2006), recalibrated into the ‘community’ units by the Nova Scotia government, were used to create community-level indices of deprivation.

### Area units and life expectancy at birth

Nova Scotia has a set of official area units called ‘communities,’ which was designed by the Nova Scotia government for the purpose of public policy development and decision making in consultation with local planning officials to better represent generally perceived community identities [24]. At the time of study, they divided the whole of the province into 276 small-areas, or communities. The area units had not been used to calculate population health statuses prior, due to their difficulty in linking any health data. Most health data contain only postal code information, and postal codes are not nested in these communities. Only in the last few years it became possible to link, with high accuracy, the address information in the Vital Statistics data to these communities using an address-based location reference file developed by a local GIS company [25]. Using these communities as a base, we created small-areas having sufficient denominator sizes (population >5,000; 5-year cumulative) needed for a stable calculation of life expectancies as recommended in literature [26]. Communities with less than 5,000 denominator for each gender were combined with other small communities that are adjacent, same settlement type (see below), and within the same counties. Twenty-five of the original communities—five parks and 20 Indian Reserves—were excluded due to very small denominator sizes and suppressed data in the Census. This procedure resulted in a total of 180 communities and community groups (herein called communities). Chiang method [27] was employed to calculate life expectancy at birth for females and males in each of the 180 communities.

### Settlement types (or rurality)

The community settlement types were determined by a process involving two steps. First, each of the original 276 communities were classified into several groups based on per km road density using geometric intervals. Geometric intervals are a statistical classification method which minimizes within group variances [28], making each group as homogeneous and unique against each other as possible. Second, a community map describing the classification was overlaid with the satellite image on Google Earth in order to determine the typologies of the classes with types of settlements. These two steps were repeated to see how many groupings would make most sense to distinguish the settlement types, resulting in seven. These classes were named based on the settlement forms and location as the following:

1. Metro

2. Suburb

3. Big satellite town

4. Mid-size town

5. Small town

6. Village & Settlement cluster

7. Sparse settlement

### Two measures of deprivation

Two indices of community deprivation were calculated for the 180 communities, adjusted for age and sex compositions based on Nova Scotia as a standard. The indices are similar to the multiple indices of deprivation developed in Quebec, [29] which have now been commonly used and shown the associations with various health indicators in a number of studies [4,30,31]. One was of material deprivation, comprised of three variables (average individual income, proportion of people with no high school diploma, and unemployment rate). Another represented social isolation, [30] comprising of variables related to the lack of immediate social ties or fragility of the social network [32] (proportion of people living alone, people who are separated, divorced or widowed, and single parents).

### Distribution of life expectancy at birth and deprivation scores by settlement types

Mean life expectancy at birth for both females and males, and mean quintile scores of material deprivation and social isolation were calculated by the seven settlement types. Additionally, mean life expectancy was compared between settlement types considered as urban and rural within HRM.

### Statistical analyses of associations between the five settlement types and life expectancy at birth

Linear regression analyses were conducted to assess the ecological-level associations between the settlement types and life expectancy at birth for females and males. First, the settlement types were treated as a continuous variable with a gradient between most urban (metro) to most rural (sparse settlements). While treating it as continuous, the gradient of rurality does not measure precise, equal intervals and only shows general ranking of how rural these types are. However, it was employed for a purpose of comparison with models that treat settlement types as a series of unique categories. Next, the seven types were dichotomized into urban (metro, suburb and big towns) and rural (mid-size town, small town, village & settlement cluster, and sparse settlement). Then, each of the settlement types was examined for its associations separately. Finally, the same analyses were repeated, adjusting for the two measures of deprivation. All the statistical analyses were conducted using SAS (Cary, NC).

## Results

Community and population distribution by the settlement types and the two measures of deprivation are presented in Table 1. The table also shows the population grouped by rural and urban. According to the grouping, about 46% of the province’s population resides in a rural setting. About 30% of the province’s population resides in Halifax Metro, while about 25% reside in sparsely populated communities.

**Table 1:** Community and population distribution by rurality (settlement types and rural–urban), socioeconomic deprivation, and social isolation in Nova Scotia, 2006

	**Number of communities**	**Population**	**(%)**	**Population**	**(%)**	**Population total**	**(%)**
		**Female**		**Male**			
**Settlement types**							
Metro	14	131,065	29.97	147,024	31.32	278,089	30.67
Suburb	15	40,611	9.29	41,081	8.75	81,692	9.01
Big satellite town	14	58,194	13.31	65,786	14.01	123,980	13.67
Mid-size town	16	34,763	7.95	38,952	8.30	73,715	8.13
Small town	32	53,678	12.27	55,960	11.92	109,638	12.09
Village & settlement cluster	64	82,795	18.93	84,128	17.92	166,923	18.41
Sparse settlement	25	34,010	7.78	34,108	7.27	68,118	7.51
**Urban–rural**							
Urban	43	229,870	52.83	253,891	54.36	483,761	53.62
Rural	137	205,246	47.17	213,148	45.64	418,394	46.38
**Material deprivation**							
Lowest	36	152,233	34.99	165,173	35.37	317,406	35.18
2	36	92,891	21.35	100,164	21.45	193,055	21.4
3	36	75,597	17.37	80,960	17.33	156,557	17.35
4	36	66,957	15.39	71,790	15.37	138,747	15.38
Highest	36	47,438	10.9	48,952	10.48	96,390	10.68
**Social isolation**							
Lowest	36	78,841	18.12	79,844	17.1	158,685	17.59
2	36	54,690	12.57	56,397	12.08	111,087	12.31
3	36	77,269	17.76	80,809	17.3	158,078	17.52
4	36	54,956	12.63	57,314	12.27	112,270	12.44
Highest	36	169,360	38.92	192,675	41.25	362,035	40.13
Total	180	435,116	100.00	467,039	100.00	902,155	100.00

Data: Census of Canada (2006), Nova Scotia Community Counts.

**Figure 1:**
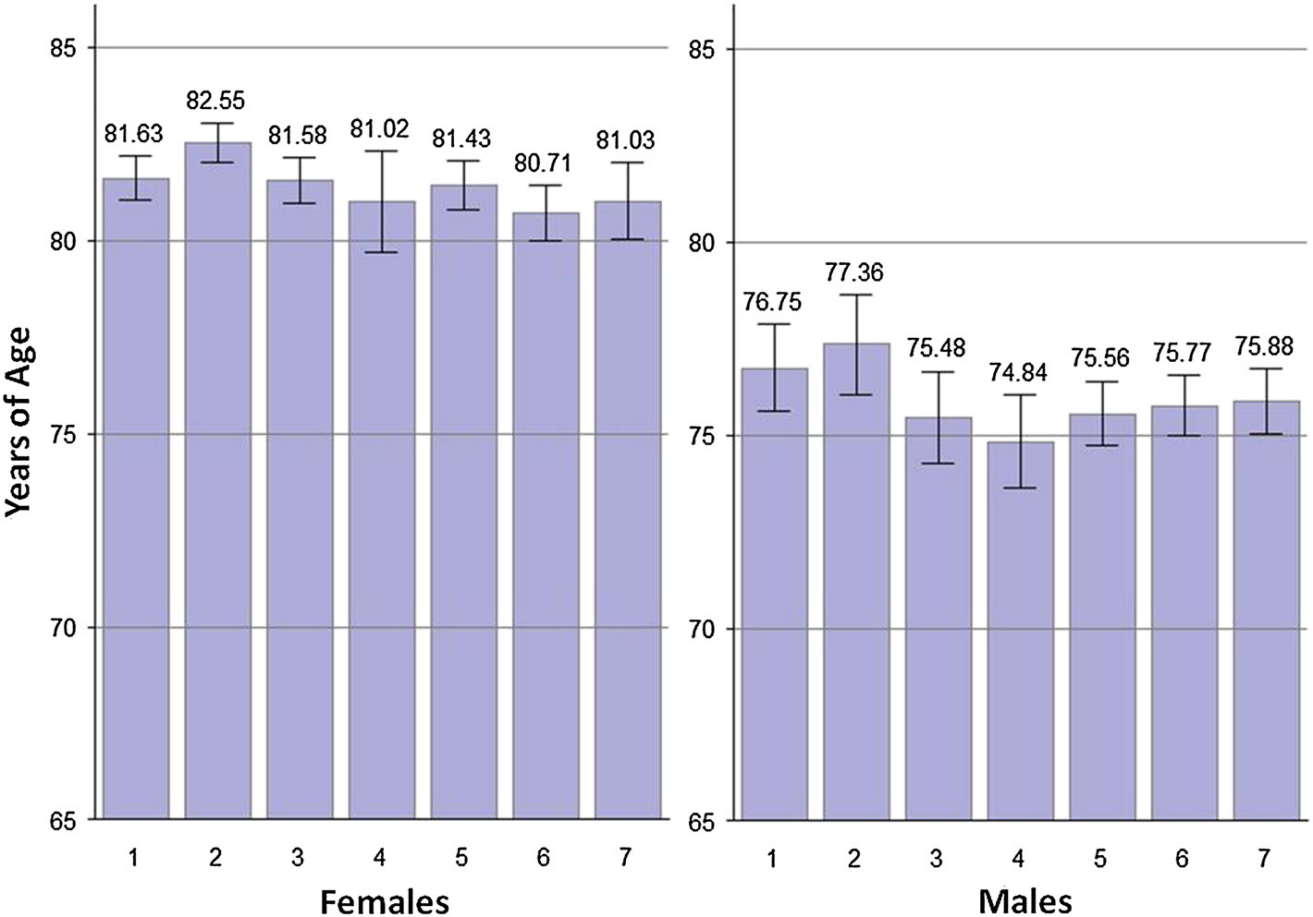
**Comparison of mean life expectancy at birth with 95% confidence intervals among seven settlement types.** 1. Metro, 2. Suburbs, 3. Big satellite town, 4. Mid-size town, 5. Small town, 6. Village & settlement cluster, 7. Sparse settlement. Data source: Nova Scotia Vital Statistics (2003–2007).

The mean life expectancy at birth for the province was 81.1 years of age for females (95% CI: 80.8, 81.5), and 75.8 years of age (95% CI:75.4, 76.21) for males. Figure 1 shows a comparison of mean life expectancy at birth for females and males among the seven settlement types. For both genders, the highest mean life expectancy was seen in suburbs, and the lowest mean was seen in the village & settlement cluster group-for females and mid-size town for males. Overall, the highest and lowest groups had 2.16 and 1.71 years of differences for females and males, respectively.

**Figure 2:**
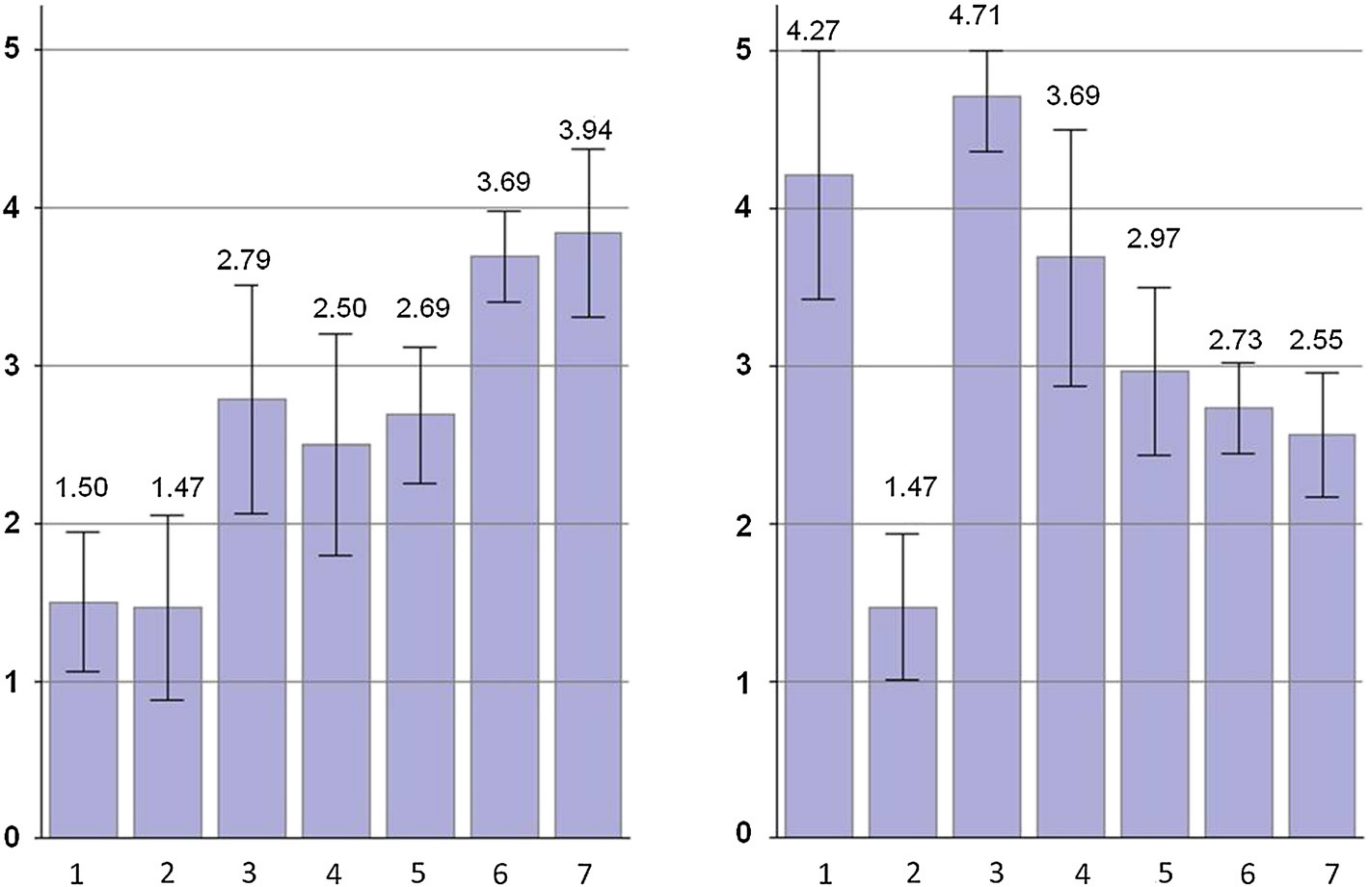
**Mean quintile score of material deprivation (left) and social isolation (right) with 95% confidence intervals among settlemen types.** 1. Metro, 2. Suburbs, 3. Big satellite town, 4. Mid-size town, 5. Small town, 6. Village & settlement cluster, 7. Sparse settlement. Data source: Census of Canada (2006), Nova Scotia Community Counts.

Figure 2 compares mean quintile scores of material deprivation and social isolation by settlement types. Affluence is clear in Metro (mean = 1.50) and suburbs (mean = 1.47), while the material condition of big satellite towns (mean = 2.79) is similar to smaller size towns (mid-size and small, mean = 2.50 and 2.69 respectively), and the two most rural settlement types were similarly deprived materially (mean = 3.69 and 3.94). Social isolation score was the highest in big satellite towns (mean = 4.71), while there appears to be general gradients of the more rural, the lower social isolation, with an exception for suburbs having a distinctly low mean score (mean = 1.47).

Before adjustment for deprivation (data not shown), the linear regression analyses indicated that the continuous measure of rural had a statistically significant, negative effect (that life expectancy was lower) for females (coefficient: -0.225; 95% CI: -0.409, -0.040), though not for males (coefficient: -0.161; 95% CI: -0.374, 0.053). The dichotomous measure of rural had a significant negative effect for both females (coefficient: -0.964; 95% CI: -1.747, -0.180) and males (coefficient: -0.912; 95% CI: -1.814, -0.010). Living in a suburb had a positive effect (that life expectancy was higher) for both females (coefficient: 1.470; 95% CI: 0.261, 2.679) and males (coefficient: 1.639; 95% CI: 0.252, 3.026), while living in a village or settlement cluster had a negative effect only on females (coefficient: -0.770; 95% CI: -1.470, -0.069).

Tables 2 and 3 show the regression models, starting with only the two deprivation measures, and then adding measures of rurality (continuous, rural–urban, and individual settlement types). The adjusted coefficient R^2^ and F-statistics indicate the proportions of variance explained by the models (with different numbers of explanatory variables) and significance of the model. Material deprivation and social isolation had statistically significant associations with life expectancy for both genders (Model 1). No interaction between material deprivation and social isolation was observed and therefore it was not included in the models. The continuous (Model 2) and dichotomous measures of rural (Model 3) remained statistically significant for females after adjusting for material deprivation and social isolation, but not for males. Adding measures of rural (Models 2 and 3) increased the proportion of variance explained slightly for females (7.6% to 10.3% and 10.0%), while it made little difference for males. The effects of living in suburbs were no longer statistically significant once adjusted by the deprivation measures (Model 4b). Only living in the village & settlement cluster type had a sustaining significant negative effect on life expectancy for females after adjusting for the two deprivation measures (Model 4e).

**Table 2:** Linear regression models on life expectancy at birth (female) and measures of rurality, adjusting for socioeconomic deprivation and social isolation, Nova Scotia 2003–2007

	**Model 1**	**Model 2**	**Model 3**	**Model 4a**	**Model 4b**	**Model 4c**	**Model 4d**	**Model 4e**	**Model 4f**	**Model 4g**
**Intercept**	83.01***	84.11***	83.59***	82.94***	82.79***	83.06***	83.01***	82.96***	83.14***	83.01***
	(82.07, 83.94)	(82.84, 85.39)	(82.54, 84.64)	(81.99, 83.89)	(81.72, 83.86)	(82.13, 83.99)	(82.07, 83.96)	(82.00, 83.92)	(82.21, 84.08)	(82.07, 83.95)
**Material deprivation**	-0.257*	-0.014	-0.091	-0.210	-0.227	-0.225	-0.260*	-0.251*	-0.146	-0.248*
	(-0.493, -0.021)	(-0.315, 0.288)	(-0.362, 0.178)	(-0.465, -0.045)	(-0.473, 0.020)	(-0.463, 0.012)	(-0.499, -0.021)	(-0.489, -0.014)	(-0.402, 0.11)	(-0.495, -0.002)
**Social isolation**	-0.349**	-0.483***	-0.439***	-0.391**	-0.321*	-0.427**	-0.346**	-0.350**	-0.412***	-0.355**
	(-0.585, -0.113)	(-0.738, -0.227)	(-0.683, -0.194)	(-0.642, -0.140)	(-0.566, -0.076)	(-0.680, -0.175)	(-0.586, -0.106)	(-0.587, -0.113)	(-0.653, -0.171)	(-0.585, -0.114)
**Rurality**		-0.298*								
		(-0.533, -0.063)								
**Urban vs rural**			-1.067*							
			(-1.953, -0.180)							
**Community types**										
Metro				0.656						
				(-0.703, 2.015)						
Suburb					0.55					
					(-0.749, 1.851)					
Big satellite town						1.099				
						(-0.204, 2.402)				
Mid-size town							-0.095			
							(-1.266, 1.076)			
Small town								0.189		
								(-0.668, 1.046)		
Settlement clusters									-0.788*	
									(-1.535, -0.042)	
Sparse settlements										-0.119
										(-1.109, 0.870)
**Adjusted R**^**2**^	0.076	0.103	0.100	0.075	0.074	0.0851	0.071	0.072	0.093	0.071
**F (Pr > F)**	8.35 (0.0003)	7.82 (<0.0001)	7.59 (<0.0001)	5.87 (0.0008)	5.79 (0.0008)	6.55 (0.0003)	5.55 (0.0012)	5.61 (0.0011)	7.12 (0.0002)	5.56 (0.0011)

*p < 0.05, **p < 0.01, ***p < 0.001.

**Table 3:** Linear regression models on life expectancy at birth (male) and measures of rurality, adjusting for socioeconomic deprivation and social isolation, Nova Scotia 2003–2007

	**Model 1**	**Model 2**	**Model 3**	**Model 4a**	**Model 4b**	**Model 4c**	**Model 4d**	**Model 4e**	**Model 4f**	**Model 4g**
**Intercept**	78.28***	78.74**	78.74***	78.16***	78.16**	78.30***	78.36***	78.40***	78.27***	78.27***
	(77.23, 79.34)	(77.29, 80.19)	(77.55, 79.93)	(77.10, 79.22)	(76.95, 79.36)	(77.24, 79.36)	(77.30, 79.41)	(77.33, 79.48)	(77.20, 79.33)	(77.21, 79.33)
**Material deprivation**	-0.373**	-0.273	-0.246	-0.287*	-0.356*	-0.365**	-0.403**	-0.387**	-0.389**	-0.388**
	(-0.639, -0.108)	(-0.617, 0.071)	(-0.553, 0.061)	(-0.573, -0.0005)	(-0.634, -0.078)	(-0.634, -0.095)	(-0.671, -0.136)	(-0.653, -0.120)	(-0.681, -0.097)	(-0.665, -0.110)
**Social isolation**	-0.443**	-0.499***	-0.513***	-0.521***	-0.427**	-0.465**	-0.407**	-0.441**	-0.435**	-0.434**
	(-0.709, -0.178)	(-0.790, -0.207)	(-0.790, -0.235)	(-0.803, -0.239)	(-0.704, -0.151)	(-0.751, -0.178)	(-0.676, -0.139)	(-0.707, -0.175)	(-0.709, -0.160)	(-0.705, -0.164)
**Rurality**		-0.123								
		(-0.392, 0.145)								
**Urban vs rural**			-0.825							
			(-1.831, 0.182)							
**Community types**										
Metro				1.214						
				(-0.309, 2.738)						
Suburb					0.323					
					(-1.143, 1.789)					
Big satellite town						0.298				
						(-1.180, 1.776)				
Mid-size town							-1.038			
							(-2.347, 0.271)			
Small town								-0.492		
								(-1.455, 0.471)		
Village & settlement cluster									0.113	
									(-0.738, 0.963)	
Sparse settlement										0.208
										(-0.906, 1.322)
**Adjusted R2**	0.108	0.107	0.116	0.116	0.104	0.104	0.115	0.108	0.1103	0.104
**F (Pr > F)**	11.85 (<0.0001)	8.16 (<0.0001)	8.84 (<0.0001)	8.79 (<0.0001)	7.93 (<0.0001)	7.92 (<0.0001)	8.78 (<0.0001)	8.24 (<0.0001)	7.88 (<0.0001)	7.91 (<0.0001)

*p < 0.05, **p < 0.01, ***p < 0.001.

Approximately 10% of the population in HRM (13 out of 41 communities) lived in communities considered as rural (Table 4). The mean life expectancy at birth for these communities was 81.45 (95% CI:79.93, 82.97) and 76.46 (95% CI: 75.49, 77.42) for females and males, respectively, while those were 82.16 (95% CI:81.76, 82.56) and 77.16 (95% CI:76.34, 77.98) for their urban counterpart.

**Table 4:** Comparisons of distribution of mean life expectancy at birth (with 95% confidence interval) by urban and rural – HRM only, rest of Nova Scotia, and Nova Scotia overall

**HRM only**		
	**Urban (n = 28)**	**Rural (n = 13)**
Females	82.16	81.45
	(81.76, 82.56)	(79.93, 82.67)
Males	77.16	76.46
	(76.34, 77.98)	(75.49, 77.43)
**Rest of Nova Scotia**		
	**Urban (n = 15)**	**Rural (n = 124)**
Females	81.52	80.92
	(80.96, 82.08)	(80.47, 81.37)
Males	75.40	75.55
	(74.29, 76.51)	(75.05, 76.05)
**Nova Scotia overall**		
	**Urban (n = 43)**	**Rural (n = 137)**
Females	81.94	80.97
	(81.61, 82.26)	(80.54, 81.40)
Males	76.55	75.64
	(75.86, 77.23)	(75.17, 76.10)

Data source: Nova Scotia Vital Statistics (2003–2007).

## Discussion

The objective of this study was to assess variations in life expectancy at birth for females and males using locally defined settlement types, at a smaller-area level than those employed in previous studies in the province. We asked whether there was an additional effect of living in rural communities other than conditions of material deprivation and social isolation, and if so, what types of communities had those effects. Additionally, we asked if there were important variations in life expectancies across communities within HRM—the most populated municipality encompassing of most urban to most rural settlement types in the province.

In general, life expectancy at birth was lower for both females and males in rural communities in Nova Scotia, which was consistent with previous studies [4,8]. The differences by settlement type were not very clear, except for suburbs being advantageous for both genders, and villages and settlement clusters being a disadvantage for females, suggesting that the impacts on health are not necessarily gradient by the level of rurality.

The health advantage of living in suburbs appears to be explained away by deprivation (i.e., suburbs being the most affluent and having the lowest level of social isolation) for both genders. One statistically significant effect of settlement type observed even after adjusting for deprivation was that of living in village and settlement clusters for females. The most rural, sparse settlement type was not associated with lower life expectancies, which suggests that there is something other than the size, remoteness or distance to amenities that produce negative effects on health in village and settlement clusters, particularly for females. To our knowledge, there is no special advantage in health service access in the most rural of communities (sparse settlements) as opposed to the village and settlement clusters in the province. The gender differentials in the effects of settlement types need to be explored further.

Although there was no statistically significant difference (i.e., there were considerable overlaps in 95% confidence intervals), mean life expectancy at birth in urban communities within HRM was about 0.7 years higher than the rural counterparts for both genders (Table 4). Moreover, the mean life expectancies of both rural and urban settlement type communities within HRM were somewhat higher than those for the rest of the province. Living in HRM appears to have an additional positive effect on health. The finding suggests that it is beneficial to investigate multi-scale of ecological (contextual) factors—community- and municipality-levels in this case.

There are some important limitations in this study. First, the study only looked at life expectancy at birth and not other health outcomes. Studies suggest that the variations in more general health statuses tend to be smaller, and a greater magnitude of variations are seen in specific diseases [4,7]. Due to its small population size, it remains a challenge for Nova Scotia to calculate different community health statuses beyond all-cause, all-age mortality at small-area level. Moreover, the use of community area units used in this study requires a better geo-reference than postal codes. Currently, there is no available health data in the province with any location information finer than postal codes, except for Vital Statistics data. Second, this is an ecological study investigating population health status and its association with settlement types of communities, adjusting for indicators of community deprivation. It does not take into account risk factors at individual level, making it impossible to clearly separate out the effects of the compositions of certain characteristics of individuals from the ‘true’ effects of the settlement types. The modest proportions (no more than 12%) of the variance in life expectancies across communities explained by the community-level factors also suggest the importance for further investigating other factors such as the clustering of individual characteristics.

What this study addressed were issues of area size and the definition of ‘rural’—which have not been dealt with adequately in many health inequality studies [4]—taking advantage of the ‘community’ area units, and detailed geo-reference data recently developed in the province. Use of areas designed to represent community entities was also an important component as they represent geographical settings in which social relations are constituted [17,33]; an issue not always addressed in the rural–urban comparison studies (or area health inequality studies in general).

## Conclusions

Detailed pictures of “serious, complex, and changing” [34] health problems in rural environments—whether in Canada or elsewhere—can only be clarified by compilation of more comprehensive studies investigating variations in health statuses and health determinants at the small-area level. In addition, regional geographical specificity has to be taken into account if diversity of rural communities were to be fully understood. Research therefore needs to find ways to overcome various data challenges in order to extend the investigation of rural–urban and within-rural health inequalities to hard-to-study provinces due to their population size. This study is a step forward to better understand the distribution of any health conditions across rural–urban continuum for a small province, using area units that are much smaller than those used in previous studies.

## Abbreviations

MIZ: Metropolitan influence zones; HRM: Halifax regional municipality; CSD: Census subdivision; CMA: Census metropolitan area; CA: Census agglomeration area.

## Competing interests

The authors declare that they have no competing interests.

## Authors’ contributions

MT designed and carried out the statistical analysis, collected the data, and drafted the manuscript. JRG participated in the revision of the paper. PA participated in the design of the study and performed some of the statistical analysis. All authors read and approved the final manuscript.

## Pre-publication history

The pre-publication history for this paper can be accessed here:

http://www.biomedcentral.com/1471-2458/14/162/prepub
